# Anti-BLV antibodies in whey correlate with bovine leukemia virus disease progression and *BoLA-DRB3* polymorphism

**DOI:** 10.3389/fvets.2022.1038101

**Published:** 2022-11-25

**Authors:** Ayumi Nakatsuchi, Aronggaowa Bao, Sonoko Watanuki, Ryosuke Matsuura, Liushiqi Borjigin, Lanlan Bai, Maho Kuroda, Yasunobu Matsumoto, Junko Kohara, Yoko Aida

**Affiliations:** ^1^Institute of Animal Health, JA Zen-Noh (National Federation of Agricultural Cooperative Associations), Sakura, Japan; ^2^Laboratory of Global Infectious Diseases Control Science, Graduate School of Agricultural and Life Sciences, The University of Tokyo, Tokyo, Japan; ^3^Laboratory of Global Animal Resource Science, Graduate School of Agricultural and Life Sciences, The University of Tokyo, Tokyo, Japan; ^4^Viral Infectious Diseases Unit, RIKEN, Wako, Japan; ^5^Agriculture Research Department, Animal Research Center, Hokkaido Research Organization, Shintoku, Japan

**Keywords:** bovine leukemia virus, anti-BLV antibodies, dam, whey, proviral load, *BoLA-DRB3*

## Abstract

**Introduction:**

Bovine leukemia virus (BLV) belongs to the family *Retroviridae* and is a causative agent for enzootic bovine leucosis, the most common neoplastic disease affecting cattle worldwide. BLV proviral load (PVL) is associated with disease progression and transmission risk but requires blood collection and quantitative PCR testing. Anti-BLV antibodies in whey have been used as a diagnostic tool for BLV infection; however, quantitative utilization has not been fully investigated. Furthermore, bovine leukocyte antigen (*BoLA*)-*DRB3* is a polymorphic gene associated with BLV infectivity and PVL, but its effect on anti-BLV antibody levels in whey from BLV infected dams is unknown. Therefore, we aimed to investigate whether it is possible to correctly predict PVL in the blood and milk based on the amount of anti-BLV antibodies in milk, and whether the *BoLA-DRB3* alleles associate with the amount of anti-BLV antibodies in milk.

**Methods:**

We examined whey from 442 dams from 11 different dairy farms located in 6 prefectures in Japan, including susceptible dams carrying at least one *BoLA*−*DRB*3^*^012:01 or ^*^015:01 allele related with high PVL, resistant dams carrying at least one BoLA-DRB3^*^002:01, ^*^009:02, or ^*^014:01:01 allele related with low PVL, and neutral dams carrying other alleles.

**Results:**

First, our results provided compelling evidence that anti-BLV antibody levels in whey were positively correlated with the anti-BLV antibody levels in serum and with BLV PVL in blood and milk, indicating the possibility of estimating BLV PVL in blood and milk by measuring anti-BLV antibody levels in whey. Thus, our results showed that antibody titers in milk might be effective for estimating BLV transmission risk and disease progression in the field. Second, we demonstrated that anti-BLV antibody levels in whey from BLV resistant dams were significantly lower than those from susceptible and neutral dams.

**Discussion:**

This is the first report suggesting that the *BoLA-DRB3* polymorphism affects anti-BLV antibody levels in whey from BLV-infected dams. Taken together, our results suggested that anti-BLV antibody levels in whey, measured by enzyme-linked immunosorbent assay, may be a useful marker to diagnose the risk of BLV infection and estimate PVL in blood and milk.

## Introduction

Bovine leukemia virus (BLV), an oncogenic member of the family *Retroviridae* and genus *Deltaretrovirus* causes enzootic bovine leukosis (EBL), the most common neoplastic disease of cattle globally (1). Approximately 70% of BLV-infected cattle are asymptomatic carriers, but approximately 30% progress to persistent lymphocytosis (PL), a condition characterized by an increased number of B lymphocytes and < 5% of cattle develop B-cell leukemia/lymphoma after a long latency period (1). BLV infection reduces milk production (2, 3) and is associated with high incidence of infectious disease (4) and reproductive inefficiency (5), resulting in high culling rates of the BLV-infected but healthy cattle (6). Therefore, BLV eradication is of outmost importance. However, owing to the lack of readily available effective treatments and vaccines, BLV infections have spread worldwide (7, 8). Currently, 40.9% of the dairy cows over 6 months of age and 28.7% of breeding cattle in Japan (9), 94.2% of dairy herds in the USA (10), 36.7% of the cattle and 78.3% of the herds in Canada (11), over 81.8% of the cattle and 99.1% of the herds in Taiwan (12), over 50% of the cattle and 86.8% of the herds in Korea (13), ~31–41.9% of the cattle in China (14, 15), 4.8–9.7% of the cattle in the Philippines (16), 21.5–28.0% of the cattle in Egypt (17, 18), and 9.1–37.04 % of the cattle in Myanmar (19, 20) are BLV positive.

BLV is transmitted primarily through the transfer of infected lymphocytes and *via* horizontal and vertical routes (1). Horizontal transmission of BLV occurs primarily *via* close contact with infected animals or *via* blood-sucking insects, such as tabanids and stable flies, (21) or *via* iatrogenic procedures, including the repeated use of individual needles, syringes, rectal palpation gloves, and dehorners (22, 23). Meanwhile, vertical transmission can include perinatal and postnatal infection. Vertical postnatal infection from cattle to calves occurs *via* colostrum and milk (24, 25); the infectious capacity of the cells in milk from BLV-infected dams is currently estimated based on the *ex vivo* visualization of BLV infection (26). In addition, there are reports of both the virus and provirus being detected in colostrum and milk (26–28), and their infectivity was confirmed by experimental inoculation and oral consumption, suggesting that there is a risk of infection through contact with these products (29). However, Konishi et al. demonstrated that the anti-BLV antibodies in the colostrum prevented BLV infection *in vitro* (30). Similarly, Porta et al. reported that when sheep were inoculated with culture supernatants containing BLV and anti-BLV antibodies at the same time, the sheep were not infected (31). However, the applicability of transferring colostrum containing anti-BLV antibodies from infected dams to prevent BLV infection in neonatal calves remains controversial.

The BLV proviral load (PVL), which represents the amount of retroviral genome integrated into the host genome, correlates strongly with disease progression (32, 33) and BLV infectivity, as assessed *via* syncytium formation (34). When PVLs exceed 10,000 copies/10^5^ cells in blood, BLV appears in the nasal secretions and saliva of cattle (35). Therefore, BLV-infected cattle with high PVLs are sources of infection for BLV-free cattle. Thus, PVL is considered a major diagnostic index for estimating BLV transmission risk (36). We previously reported the presence of BLV provirus in milk samples and showed positive association of PVL in milk with that in the blood, indicating that the PVL in milk is a useful marker of the PVL of the peripheral blood (26). However, the PVL in milk tends to be lower than that in the peripheral blood and it is more difficult to detect PVL in milk than in peripheral blood samples (26). This shows that measuring PVL in milk is difficult to use in the field conditions. By contrast, anti-BLV enzyme-linked immunosorbent assay (ELISA) values in whey closely correlate (97%) with of those in serum (37), indicating that antibody titer is a highly effective measure for using in the field. Indeed, studies with small sample sizes have shown that the anti-BLV antibody levels in colostrum or milk are positively correlated with the BLV PVL in the blood (38, 39). However, a bigger sample size is needed to conclusively establish a correlation between anti-BLV antibodies in milk (not in colostrum) and PVL in the peripheral blood. In addition, whether anti-BLV antibodies in milk are useful markers for the detection of anti-BLV antibodies in blood and PVL in milk remains unclear.

PVL and anti-BLV antibody production levels are strongly associated with the highly polymorphic bovine leukocyte antigen (*BoLA*)-*DRB3* gene (40–43). In total, 384 alleles are registered in the Immuno Polymorphism Database (IPD)-MHC database (https://www.ebi.ac.uk/ipd/mhc/group/BoLA/, accessed on 2 September 2022). Previous association studies demonstrated that the BoLA-DRB3^*^015:01 and DRB3^*^012:01 alleles are susceptibility-associated markers related to high PVL levels (41), while the BoLA-DRB3^*^009:02, DRB3^*^014:01:01 (40, 41, 43, 44), and DRB3^*^002:01 alleles (43) are resistance markers associated with the development of low PVL. Other *BoLA-DRB3* alleles were not significantly associated with PVL *in vivo* (40). Thus, *BoLA-DRB3* alleles are believed to determine cattle–specific differences related to resistance to BLV and disease progression. However, whether polymorphisms in *BoLA-DRB3* alleles are related to the amount of anti-BLV antibodies in milk remains unknown.

In this study, we investigated whether it is possible to accurately predict BLV PVLs in the blood based on the amount of anti-BLV antibodies in milk, which can be collected non-invasively. First, the cut-off points for the ELISA using milk samples were calculated from a receiver operating characteristic curve (ROC) derived from the milk samples of 259 dams. Second, the associations between anti-BLV antibodies in milk and PVL in blood, and anti-BLV antibodies in blood and PVL in milk were clarified. Finally, the association between the *BoLA-DRB3* allele and the amount of anti-BLV antibodies in milk was investigated. Our results provide evidence that the levels of anti-BLV antibody in milk, as determined by ELISA, may be a useful marker to diagnose the risk of BLV transmission and to estimate PVLs in blood and *BoLA-DRB3* polymorphism regulates the anti-BLV antibody level in whey may be an effective for breeding strategy.

## Materials and methods

### Cattle background, collection of milk and blood samples, and genomic DNA extraction

From 2012 to 2021, milk and blood samples were collected from 442 Holstein-Friesian dams without lymphoma, from 11 different dairy farms located in Hokkaido, Gunma, Tochigi, Saitama, Kanagawa, and Chiba prefectures in Japan (Table 1). This study was approved by the animal ethical committee, and the animal care and use: RIKEN animal experiments committee (Approval Number H29–2–104).

**Table 1:** Bovine leukemia virus (BLV) prevalence and sample number and for each farm.

**Farm**	**Total dams**	**BLV+ dams^a^**	**Sample number**			
			**Milk**		**Blood**	
			**whey**	**DNA**	**serum^b^**	**DNA**
A	51	51	51	0	44	46
B	49	49	49	0	47	48
C	74	74	74	0	74	73
D	40	34	40	0	0	0
E	17	13	17	0	0	0
F	11	9	11	8	9	9
G	12	12	12	12	8	12
J	4	4	4	4	4	4
H	10	10	10	10	10	10
I	15	15	15	15	15	15
K	159	0	159	0	0	0
Total	442	271	442	49	211	217

^a^BLV+ was determined by a serum-based anti-BLV antibody ELISA (Nippon Gene, Tokyo, Japan).

^b^Blood samples were collected from 442 Holstein-Friesian dams and serum was collected from blood from all dams, and the 211 of them were used for correlation analysis between anti-BLV antibody in serum and whey and determination of cut-off values for anti-BLV antibody ELISA using whey.

Serum was collected from all dams and BLV infection was confirmed using anti-BLV antibody ELISA kit (Nippon Gene, Tokyo, Japan) according to the manufacturer's instructions. The 211 of them were used for correlation analysis between anti-BLV antibody in serum and whey and determination of cut-off values for anti-BLV antibody ELISA using whey (Table 1). Whey was obtained from the milk of all dams by centrifugation at 4,000 × g for 3 min to detect anti-BLV gp51 antibody (Table 1). Serum and whey were stored at −20°C.

For the measurement of PVL in milk, genomic DNA extraction from milk was collected from 48 dams in five farms, as previously described (26) (Table 1). Briefly, 100 mL milk was collected, maintained at 4°C, and then transported to the laboratory within 24 h. Milk samples were transferred to 50 mL sterile tubes and centrifuged at 4,000 × g for 15 min to remove the cream layer, protein, and whey. The pellets, including milk cells and the remaining supernatant, were transferred to 15 mL sterile tubes and centrifuged at 1,000 × g for 30 min. The pellets were resuspended in 15 mL phosphate-buffered saline (PBS) and washed twice at 1,600 rpm for 5 min and 1,200 rpm for 5 min. DNA was extracted from the pellets using a Wizard Genomic DNA Purification Kit (Promega Corporation, Madison, WI, USA) with 1.54 mg/mL of dithiothreitol, according to the manufacturer's instructions. For the measurement of the BLV PVL in blood, blood samples were obtained from 217 Holstein-Friesian dams infected with BLV and stored in ethylenediaminetetraacetic acid (EDTA) (Table 1).

DNA was extracted from the blood samples using the Wizard^®^ Genomic DNA Purification Kit (Promega corporation) according to the manufacturer's instructions. The genomic DNA concentration was measured using a Nanodrop 2000c spectrophotometer (Thermo Fisher Scientific, Waltham, MA, USA). DNA samples were stored at −20°C.

### Determination of cut-off values for the anti-BLV antibody ELISA using whey

Whey from 88 BLV-positive dams and 171 BLV-negative dams was used to determine the cut-off value for anti-BLV antibody ELISA. Anti-BLV gp51 antibodies in whey were measured using an anti-BLV antibody ELISA kit (Nippon Gene) according to the manufacturer's instructions. Optical density (OD) values were read, and the status of the evaluated samples was calculated as the ratio of antigen presence in the sample to that in the positive control (S/P ratio) as follows:


$$\frac{S}{P}=\frac{Sample\left(antigen-no\;antigen\right)-Blank}{Positive\;control\;(antigen-no\;antigen)-Blank}$$

The cut-off value for the S/P ratio was calculated from receiver operating characteristics (ROC) curves.

### Determination of anti-BLV antibody value in whey and serum

Anti-BLV gp51 antibodies were measured using an anti-BLV antibody ELISA kit (Nippon Gene). Briefly, 32-fold dilution of whey and 512-fold dilution of serum were used. ELISA was performed with diluted whey and serum according to the manufacturer's instructions. OD values were read, and the status of the samples evaluated by the S/P ratio was calculated: those samples with reactivity above the cut-off value were considered positive. Anti-BLV gp-51 antibody titers were determined by performing ELISA on 2-fold step dilutions of whey and serum. Titers were expressed as the reciprocal of the last dilution with reactivity above the cut-off value. Titers were expressed as the binary logarithm of the last dilution that showed reactivity above the cut-off value.

### Evaluation of the BLV PVL by BLV-CoCoMo-qPCR-2 in milk and blood

BLV PVL in milk and blood samples was determined using the BLV-CoCoMo-qPCR-2 assay (33, 45, 46) with THUNDERBIRD Probe qPCR Mix (Toyobo, Tokyo, Japan). Amplification was performed using the Light Cycler^®^ 480 System II (Roche Diagnostics, Mannheim, Germany) or the QuantStudio^®^ 5 Real-Time PCR System (Thermo Fisher Scientific). PVL was estimated as the copy number in 10^5^ white blood cells or milk cells.

### *BoLA-DRB3* allele typing

*BoLA-DRB3* was identified using the polymerase chain reaction (PCR)-sequence-based typing (SBT) method using genomic DNA from blood, as previously described (47). Briefly, *BoLA-DRB3* exon 2 was amplified using PCR with DRB3FRW and DRB3 REV primers. The PCR fragments were purified and sequenced using the BigDye™ Terminator v1.1 Cycle Sequencing Kit (Thermo Fisher Scientific). Sequencing data were analyzed to determine *BoLA-DRB3* alleles using the ASSIGN 400 AFT software (Conexio Genomics, Fremantle, Australia).

### Statistical analysis

The ROC analysis was performed using the R package, version 4.1.2, to determine the cut-off value of anti-BLV antibody level in whey. The correlation coefficient (*r*) was calculated using the Pearson function in Excel. Tukey's multiple comparison test was used to determine the statistical significance of the differences in the PVL measured in the milk obtained from the susceptible, neutral, and resistant groups. A *p* < 0.05 was considered statistically significant.

## Results

### Determining the cut-off point for the BLV ELISA using whey

**Figure 1:**
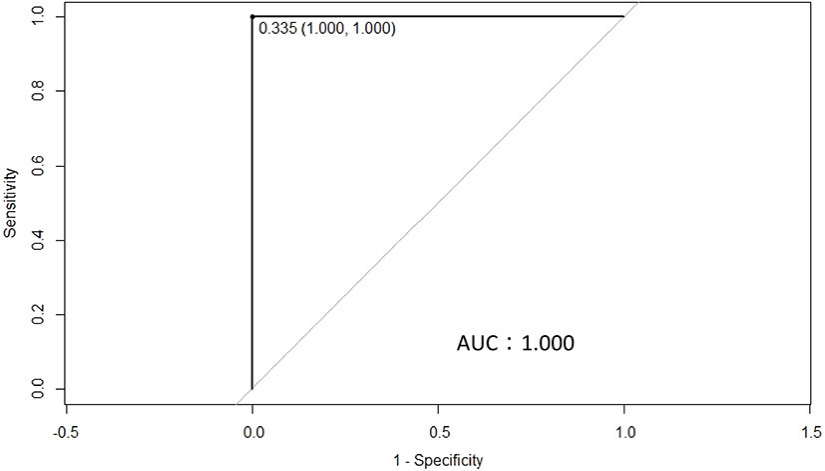
A receiver operating characteristics (ROC) curve was used to calculate the cut-off point for the S/P ratios of ELISA using whey from 259 dams that were either positive (88 dams) or negative (171 dams) for bovine leukemia virus (BLV), as determined by ELISA in serum. The cut-off point for the S/P ratio was 0.335, while the area under the curve was 1.000.

To determine the differences in anti-BLV antibody levels in the whey of BLV-infected dams, we determined the cut-off value for anti-BLV gp51 ELISA using whey. First, whey from 442 Holstein-Friesian dams was collected at 11 different dairy farms in Japan (Table 1). Serum was also collected from all dams to confirm the BLV infection using the anti-BLV antibody ELISA kit. Next, whey from 88 BLV-positive dams and 176 BLV-negative dams out of 442 dams was selected and ROC curves were constructed to evaluate the potential of antibodies in discriminating between animals with a detectable and those with an undetectable level of anti-BLV antibody in whey (Figure 1). ROC curve analysis yielded an area under the curve (AUC) of 1.000, which enables meaningful interpretation of anti-BLV antibody levels in whey in BLV-positive and -negative cattle. Here, the cut-off for the S/P ratio was calculated to be 0.335 when whey was used.

### Positive correlation between anti-BLV antibody levels in whey and serum

**Figure 2:**
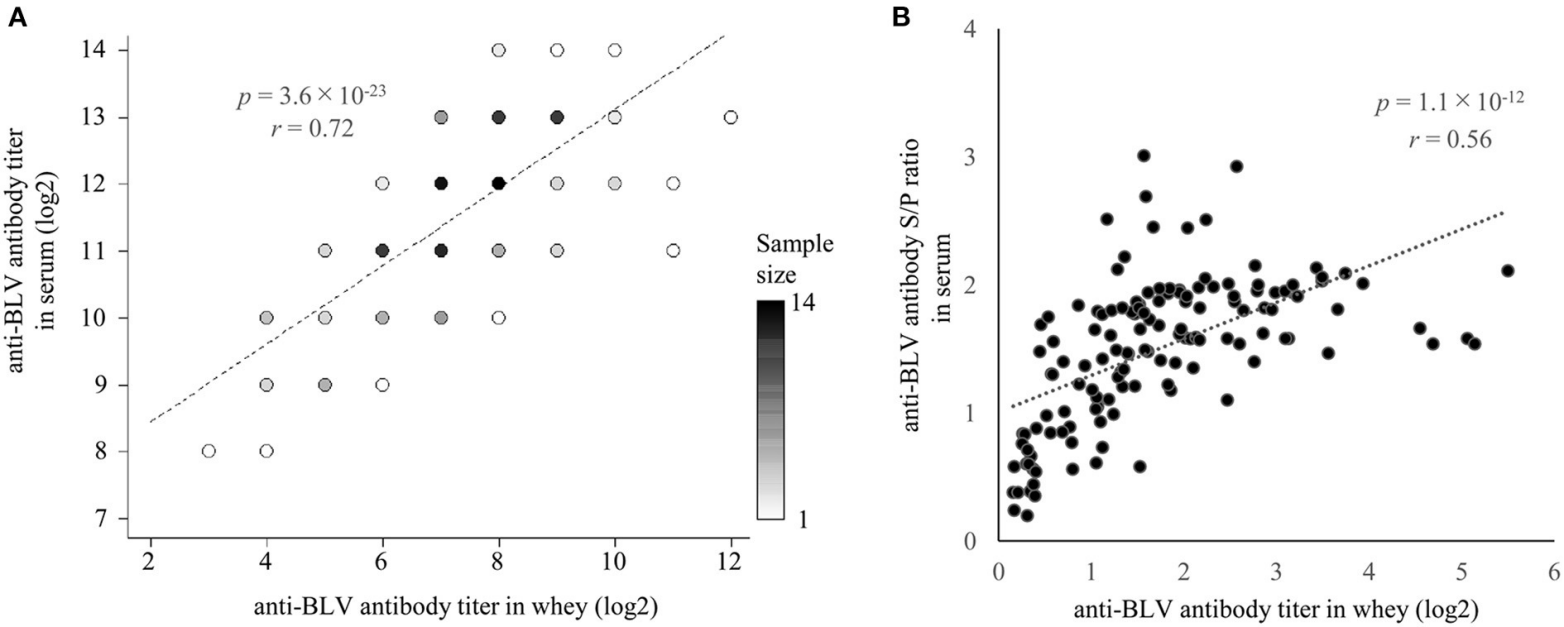
The correlation between anti-bovine leukemia virus (BLV) antibody presence in whey and serum. Anti-BLV antibody titers, S/P ratios at 32-fold dilution in whey, and S/P ratios at 512-fold dilution in serum were measured using an anti-BLV antibody ELISA. **(A)** Correlation between anti-BLV antibody titer in whey and serum (*n* = 137). Dot color shows sample size. **(B)** Correlation between anti-BLV antibody S/P ratios for 32-fold dilution in whey and 512-fold dilution in serum (*n* = 137).

Previously, Jaworski et al. reported that anti-BLV antibody titers in whey were positively correlated with those in serum (38). On the other hand, Konishi et al. reported no correlation between anti-BLV antibody titers in colostrum and those in serum (30). Since both reports used < 50 samples, we used one set of whey and serum from 137 dams and compared the anti-BLV antibody levels in each (Figure 2). As a result of statistical analysis, although the anti-BLV antibody titer in whey was 4–32 times lower than that in the serum, it showed a positive correlation with the anti-BLV antibody titer contained in serum (*r* = 0.72, *p* = 3.6 × 10^−23^) (Figure 2A). Similarly, S/P ratios of anti-BLV antibodies in the 32-fold diluted sample of whey showed a positive correlation with the 512-fold diluted serum sample (*r* = 0.56, *p* = 1.1 × 10^−12^) (Figure 2B). Our results showed that the anti-BLV antibody levels in whey of dams naturally infected with BLV was positively correlated with the anti-BLV antibody levels in serum.

### A positive correlation between anti-BLV antibody levels in whey and BLV PVL in blood

**Figure 3:**
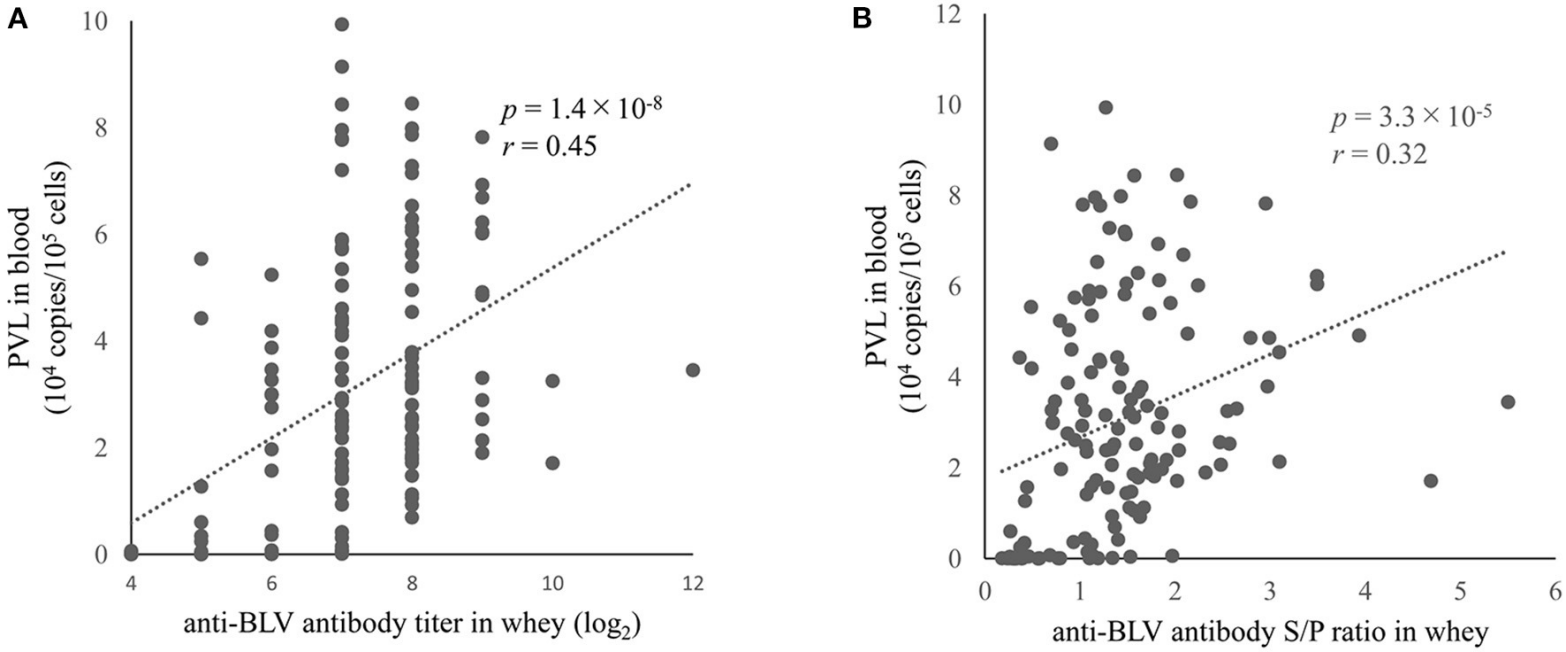
The correlation between anti-bovine leukemia virus (BLV) antibody in whey and BLV proviral load (PVL) in blood. Anti-BLV antibody titers and S/P ratios for 32-fold dilution in whey were measured using an anti-BLV antibody ELISA. The PVLs in blood were measured using the CoCoMo-qPCR-2 method (RIKEN Genesis, Kanagawa, Japan). **(A)** The correlation between anti-BLV antibody titer in whey and BLV PVL in blood (*n* = 145). **(B)** Correlation between anti-BLV antibody S/P ratios at 32-fold dilution in whey and BLV PVL in blood (*n* = 145).

PVL in blood is considered a major diagnostic index for estimating BLV transmission risk (36) and as a risk factor for the progression of EBL (32, 33). Therefore, it is important to clarify whether it is possible to correctly predict PVL in blood based on the amount of anti-BLV antibodies in milk, which is easy to collect. Indeed, previous reports have shown that the anti-BLV antibody level in colostrum (30) or milk (38), is positively correlated with the BLV PVL in blood. However, the previous two studies were limited by < 50 samples subjected to the experiment. Therefore, here we measured BLV PVL using DNA in the blood from 145 dams. The results showed that BLV PVL in the blood of these 145 dams was positively correlated with the anti-BLV antibody titers in the whey (*r* = 0.45, *p* = 1.4 × 10^−8^) (Figure 3A). Furthermore, a comparison of the S/P ratios of anti-BLV antibodies in 32-fold diluted samples of whey and BLV PVL in blood showed a weak positive correlation (*r* = 0.32, *p* = 3.3 × 10^−5^) (Figure 3B). Our results showed that the anti-BLV antibody levels in whey were positively correlated with the amount of BLV PVL in blood.

### A positive correlation between anti-blv antibody levels in whey and BLV PVL in milk

**Figure 4:**
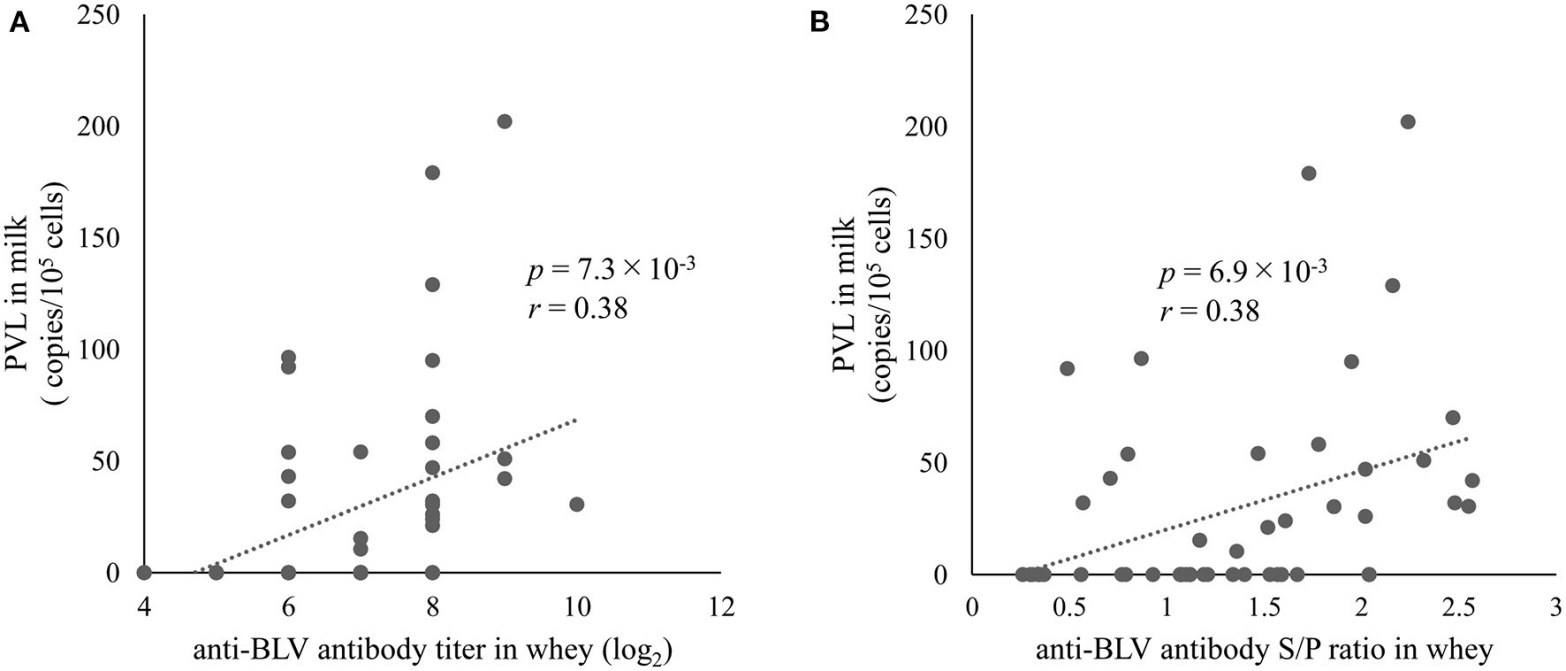
The correlation between anti-bovine leukemia virus (BLV) antibody in whey and BLV proviral load (PVL) in milk. Anti-BLV antibody titers and S/P ratios at 32-fold dilution in whey were measured using an anti-BLV antibody ELISA. The PVLs in milk were measured using the CoCoMo-qPCR-2 method (RIKEN Genesis, Kanagawa, Japan). **(A)** The correlation between anti-BLV antibody titer in whey and BLV PVL in milk (*n* = 49). **(B)** The correlation between anti-BLV antibody S/P ratios at 32-fold dilution in whey and BLV PVL in milk (*n* = 49).

We found a positive correlation between the anti-BLV antibody levels in whey and BLV PVL in blood as mentioned above. In addition, we previously showed a positive correlation between BLV PVL in blood and milk (26, 48), indicating that anti-BLV antibody titers in whey may positively associate with BLV PVL in milk. However, Jaworski et al. reported that anti-BLV antibody titers in whey and BLV PVL in milk were negatively correlated (38). Therefore, we evaluated the relationship between anti-BLV antibody levels and BLV PVL in milk from 49 dams. The results showed that BLV PVL in milk showed a weak positive correlation with the anti-BLV antibody titer in the whey of those dams (*r* = 0.38, *p* = 7.3 × 10^−3^) (Figure 4A). Similarly, a comparison of S/P ratios of anti-BLV antibodies in the 32-fold diluted sample of whey and BLV PVL in milk showed a weak positive correlation (*r* = 0.38, *p* = 6.9 × 10^−3^) (Figure 4B). These results indicate that the anti-BLV antibody levels in whey is positively correlated with BLV PVL in milk.

### A positive relationship between the anti-BLV antibody levels in whey with the anti-BLV antibody levels in blood, BVL PVL in blood and milk

As summarized in Table 2, our results indicated that the anti-BLV antibody titer in whey was positively correlated with the anti-BLV antibody titer in blood, BVL PVL in blood and milk. Likewise, S/P ratios of anti-BLV antibodies in the 32-fold diluted samples of whey also showed a positive correlation with both S/P ratios of the anti-BLV antibody in the 512-fold diluted sample of serum and the BVL PVL in blood and milk, but the correlation coefficient tended to be lower than that of the anti-BLV antibody titers.

**Table 2:** Positive correlation between the amount of anti-BLV antibodies in whey and the amount of anti-BLV antibodies in serum, BLV PVL in blood, and BLV PVL in milk.

	**Anti-BLV antibody in whey**	
	**Titer**	**S/P ratio**
Anti-BLV antibody in serum	Positive correlation (*r* = 0.72)	Positive correlation (*r* = 0.56)
BLV PVL in blood	Positive correlation (*r* = 0.45)	Positive correlation (*r* = 0.32)
BLV PVL in milk	Positive correlation (*r* = 0.38)	Positive correlation (*r* = 0.38)

### Detection of anti-BLV antibody levels in the whey of BLV susceptible, neutral, and resistant dams from different dairy farms

**Figure 5:**
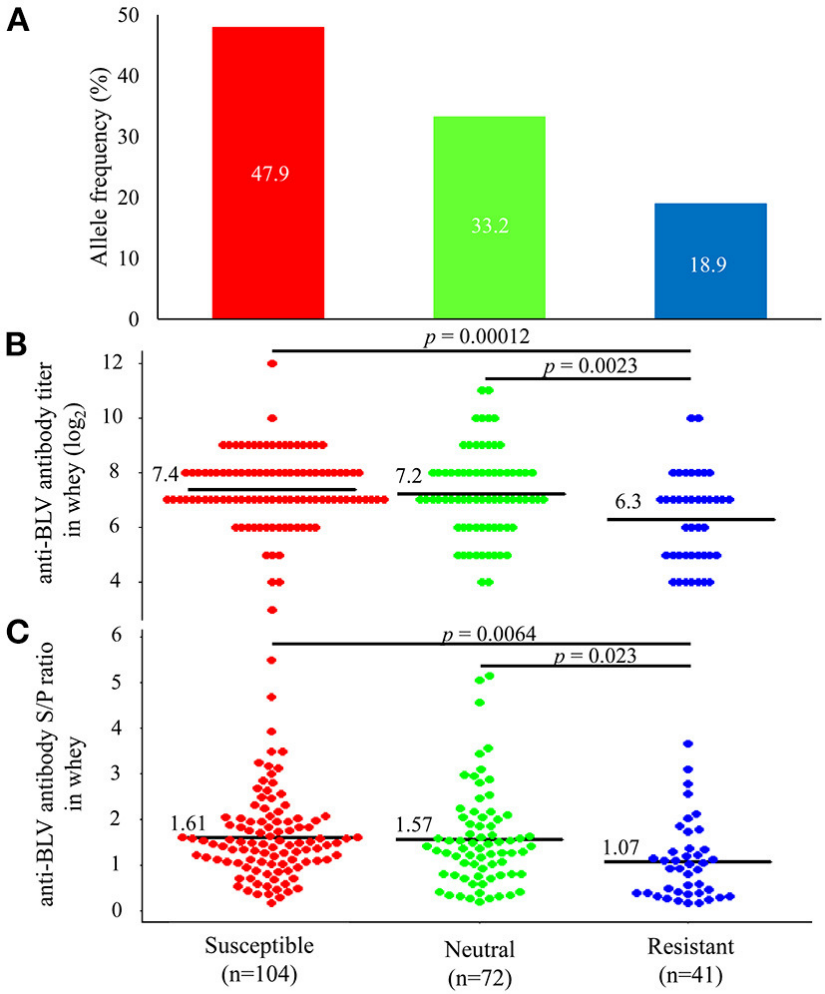
Differences in the amount of anti-bovine leukemia virus (BLV) antibodies in whey from susceptible, resistant, and neutral dams. **(A)** Allele frequency of *BoLA-DRB3* in 217 BLV-positive dams. *BoLA-DRB3* allele were typed by the PCR-SBT method using DNA from blood from 217 BLV-positive dams. All dams were divided into susceptible, neutral, and resistant dam groups based on the *BoLA-DRB3* allele status, as follows: susceptible dams carried at least one susceptible *BoLA-DRB3* *012:01 or *015:01 allele (red) but did not carry resistant alleles; resistant dams carried at least one resistant BoLA-DRB3*002:01, *009:02, or *014:01:01 allele (blue); and neutral dams carried other alleles (green) in their genomes **(B,C)** Anti-BLV antibody titers **(B)** and S/P ratios at 32-fold dilution **(C)** in whey for each group. Anti-BLV antibody titers and S/P ratios at 32-fold dilution in whey were measured using an anti-BLV antibody ELISA. Mean anti-BLV antibody titers and S/P ratios among groups were compared using Tukey's multiple comparison test.

Previous studies have reported that *BoLA-DRB3* is associated with BLV PVL in blood and milk (26, 40, 41, 43, 44, 48–51). Therefore, to investigate the relationship between the anti-BLV antibody levels in the whey and *BoLA-DRB3*, we first collected blood from 217 cattle at 8 different dairy farms in Japan (Table 1) and typed the *BoLA-DRB3* alleles using PCR-SBT method. PCR-SBT identified 14 known *BoLA-DRB3* alleles (BoLA-DRB3^*^001:01, ^*^002:01, ^*^006:01, ^*^007:01, ^*^009:02, ^*^010:01, ^*^011:01, ^*^012:01, ^*^014:01:01, ^*^015:01, ^*^016:01, ^*^017:01, ^*^018:01, and ^*^027:03) in the IPD-MHC database that were used to divide the dams into BLV susceptible, resistant, and neutral groups. BLV susceptible dams carried at least one susceptible BoLA-DRB3^*^012:01 or ^*^015:01 allele and were associated with high BLV PVL. BLV resistant dams carried at least one resistant BoLA-DRB3^*^002:01, ^*^009:02, or ^*^014:01:01 allele and were associated with low BLV PVL (40, 41, 43, 44). Dams carrying both resistance and susceptible alleles were defined as BLV resistant because the resistance trait was more dominant than the susceptibility one (52). BLV neutral dams carried other alleles. The number of susceptible, neutral, and resistant dams was 104 (47.9%), 72 (33.3%), and 41 (18.9%), respectively (Figure 5A). Next, whey from the same Holstein-Friesian dams was collected (Table 1) and used to determine the anti-BLV antibody levels in each sample.

### Anti-BLV antibody levels in the whey of resistant dams were significantly lower than in those of the other dams

Comparison of anti-BLV antibody levels in whey from 104 susceptible dams, 72 neutral dams, and 41 resistant dams showed that the average anti-BLV antibody titer in whey was 7.4, 7.2, and 6.3 for the susceptible, neutral, and resistant groups, respectively (Figure 5B). Moreover, the average anti-BLV antibody titer in whey from the susceptible and neutral groups were significantly higher than those from the resistant group (*p* = 0.00012 and *p* = 0.0023). Furthermore, mean anti-BLV antibody S/P ratio in whey was 1.61, 1.57, and 1.07 for the susceptible, neutral, and resistant groups, respectively (Figure 5C). These results indicated that the anti-BLV antibody S/P ratio in the whey of susceptible and neutral dams was significantly higher than that in the whey of resistant dams (*p* = 0.0064 and *p* = 0.023).

### Genotype of *BoLA-DRB3* allele is associated with anti-BLV antibody levels in milk

**Figure 6:**
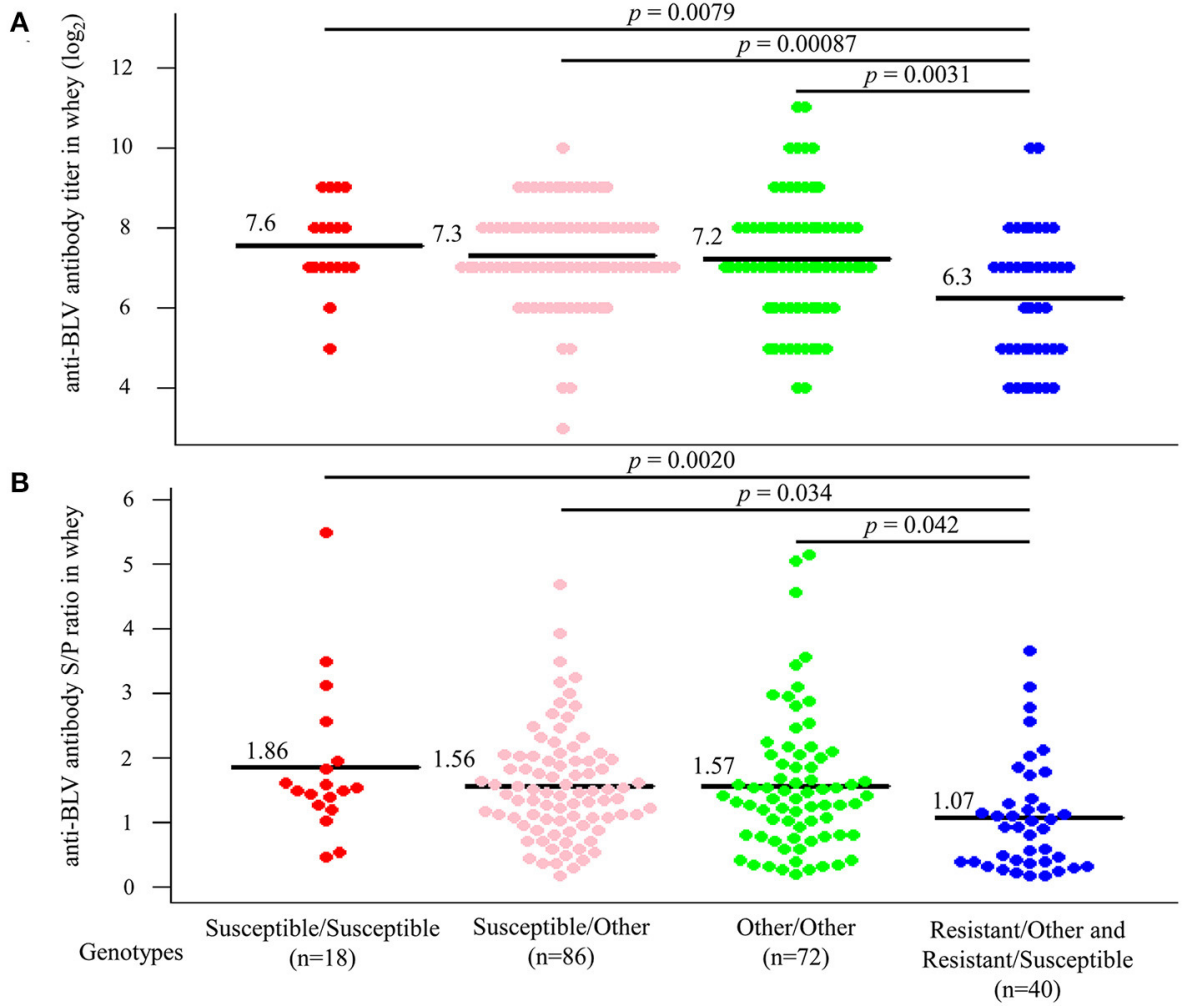
Differences in the amount of anti-bovine leukemia virus (BLV) antibodies in whey according to genotypes. All dams were divided into Susceptible/Susceptible, Susceptible/Other, Other/Other, and Resistant/Other or Susceptible based on genotypes. **(A,B)** Anti-BLV antibody titers **(A)** and S/P ratios at 32-fold dilution **(B)** in whey for each genotype. Anti-BLV antibody titers and S/P ratios at 32-fold dilution in whey were measured using an anti-BLV antibody ELISA. Mean anti-BLV antibody titers and S/P ratios among groups were compared using Tukey's multiple comparison test.

Next, dams were divided into four groups based on whether their genotypes were homozygous or heterozygous for the susceptible allele. As for the resistant allele, only one dam homozygous for the resistant allele was excluded from the statistical analysis. Anti-BLV antibody titer in whey was compared based on each genotype (Figure 6A). The results indicated that 18 dams that carried a homozygous genotype for susceptible alleles had the highest mean anti-BLV antibody titer in whey (7.6), followed by 86 dams with a heterozygous genotype for a susceptible allele with another allele (7.3), 72 dams with a homozygous genotype for another allele (7.2), and 40 dams with a heterozygous genotype for a resistant allele with another allele or susceptible allele (6.3). Moreover, the average anti-BLV antibody titer in whey from the susceptible/susceptible, susceptible/other, and other/other groups were significantly higher than that from the resistant group (*p* = 0.0079, *p* = 0.00087, and *p* = 0.0031, respectively). Similarly, anti-BLV antibody S/P ratio in whey was compared based on each genotype (Figure 6B). The susceptible/susceptible group had the highest mean anti-BLV antibody S/P ratio in whey (1.86), and resistant/other and resistant/susceptible group had the lowest mean anti-BLV antibody S/P ratio in whey (1.07). The average anti-BLV antibody S/P ratio in whey from the susceptible/susceptible, susceptible/other, and other/other groups were significantly higher than that from the resistant group (*p* = 0.0020, *p* = 0.034, and *p* = 0.042, respectively).

Thus, anti-BLV antibody levels in whey were the highest in the milk from dams possessing a homozygous genotype for susceptible alleles, while it was the lowest in the milk from dams possessing a heterozygous genotype for resistant alleles.

## Discussion

There is substantial literature on assessing the risk of BLV transmission based on the measure of BLV PVL in blood (24, 35, 51, 53). However, for measuring the BLV PVL in blood, the services of a trained veterinarian are required for drawing the blood from the animal, and the cost of analysis is higher as compared with the cost incurred for the detection of viral antibodies in milk. In this context, in the present study, we investigated the utility of anti-BLV antibody titer in whey of infected dams for estimating disease risk and transmission. First, we demonstrated that it is possible to estimate the BLV PVL in the blood and milk based on anti-BLV antibody levels in the whey detected by ELISA, which is widely used to diagnose BLV infection due to the ease of the collection of the required samples and its non-invasiveness. Our results provided strong evidence that anti-BLV antibody levels in whey were positively correlated with the anti-BLV antibody levels in serum and with BLV PVL in blood and milk. The correlation coefficient between whey and serum anti-BLV antibody titers was 0.72, which is a higher correlation than previously reported (38). Anti-BLV antibody levels in whey also proved to be positively correlated with BLV PVL in blood. The correlation coefficient (*r* = 0.45) between anti-BLV antibody titer in whey and BLV PVL in blood was lower than that between anti-BLV antibody levels in whey and serum. However, these correlation coefficients are consistent with the results stated from a published study that confirm the correlation (38). Contrary to previous reports (30, 38), to the best of our knowledge this is the first study to find that anti-BLV antibody levels in whey were also positively correlated with BLV PVL in milk. These correlation coefficients were similar to those between anti-BLV antibody levels in whey and BLV PVL in blood. The reason for this conflicting finding is to considered be the fact that higher BLV PVL in the blood and milk causes an increase in anti-BLV antibody production in milk (38, 43). To increase the precision of results, future studies incorporating large numbers of milk samples are urgently needed. Thus, our current data show that it is possible to estimate BLV PVL in blood and milk by measuring the anti-BLV antibody titer in whey, and in addition, antibody titer in milk may be effective for estimating BLV transmission risk and disease progression in the field. On the other hand, previous studies have shown that anti-BLV antibody levels in the serum of infected test cattle and naturally infected cattle differed significantly depending on *BoLA-DRB3* polymorphism (43, 54). Secondly, we here found that dams carrying the resistance allele had significantly lower levels of anti-BLV antibodies in whey compared with the dams carrying the other alleles. The anti-BLV antibody titer in relation to the *BoLA-DRB3* polymorphism showed the following order: susceptible > neutral > resistant dams. Dams homozygous for the susceptibility allele had the highest levels of anti-BLV antibodies in whey compared with dams with other genotypes. These results provide the first evidence that *BoLA-DRB3* polymorphism affects anti-BLV antibody levels in whey and are consistent with those of previous studies indicating that *BoLA-DRB3* affects BLV PVL in milk (48).

Previously it has been shown that the PVL in milk tends to be in lower amount than that in the peripheral blood, thus, it is more difficult to detect PVL in milk compared to peripheral blood samples (26), indicating that DNA extraction from milk requires a large amount of milk sample. Therefore, in this study, milk samples from some cows could not be obtained due to insufficient cooperation from farmers and farmer's convenience at the time of sampling. In addition, it was not feasible to collect milk from several cows because of a dry period and mastitis. Thus, in this study, the number of milk samples tested for PVL is less than that of blood samples, consequently the precision of results may vary. Therefore, it is necessary to increase the number of DNA samples from milk in the future to accurately evaluate the proposed relationship with anti-BLV antibody levels in whey.

Konishi et al. reported no significant correlation between the anti-BVL antibody titers in colostrum and those in serum and the PVL in colostrum (30). In contrast, we used milk in this study and demonstrated that there was a significant correlation between the anti-BVL antibody titers in milk and the antibody titers in serum and the BLV PVL in milk. In addition, Jaworski et al. reported a negative correlation between anti-BLV antibody levels in whey and BLV PVL in milk (38). In contrast, our results showed a positive correlation. One plausible explanation for this difference could be the sample size and the methods used to measure BLV PVL in milk. One previous study used colostrum and serum from 50 dams to evaluate the relationship between the antibody titers in each, while our experiment used whey and serum from 137 dams. By more than doubling the sample size, we believed that we were able to assess anti-BLV antibody levels in whey and serum with greater precision than was obtained in previous studies (30, 38). Further, in previous studies, BLV PVL was measured per 10 ng DNA or per ng DNA, and BLV PVL per cell in milk was not calculated. In our experiment, to normalize the viral genomic DNA, our BLV-CoCoMo-qPCR technique amplified a single-copy of the host gene, the *BoLA-DRA* gene, in parallel with the viral genomic DNA (33, 45, 46). Therefore, the BLV PVL per cell in milk was calculated and then evaluated in relation to anti-BLV antibody levels in whey, which may have caused the difference with the experimental results of previous studies.

The collection of whey is an easy and non-invasive method of sample collection as compared to blood sample collection, which is invasive and requires a veterinarian and the use of expensive medical materials. Our results indicate that whey can be used not only to diagnose BLV infection, but also to estimate BLV PVL in blood and milk; therefore, the anti-BLV antibody titer in whey can be considered a major diagnostic index for the estimation of BLV transmission risk and disease progression. In addition, our research will provide farmers with a simpler, less expensive, and more animal welfare-compliant way to monitor on-farm BLV transmission. A previous study reported that perinatal transmission risk was markedly low for dams and calves carrying resistant alleles as compared with those harboring susceptible alleles, thereby reducing the risk of vertical transmission (53). In addition, it was demonstrated that BLV-infected susceptible dams were at high risk of developing severe infection and showing high PVL in blood as compared with resistant dams, who showed low infection severity and low PVL in blood. Thus, the preferential culling of cattle with susceptible *BoLA-DRB3* alleles could reduce the risk of both horizontal and vertical transmission (51). Interestingly, our results showed that dams carrying the resistance allele had significantly lower levels of anti-BLV antibodies in whey than those carrying susceptible alleles. In addition to the effectiveness of assessing the presence of the resistant or susceptible alleles in cattle for BLV eradication, the measurement of anti-BLV antibody levels in whey is expected to make it possible to estimate on-farm infection status and individual PVL in blood and milk, which in turn will help to determine the risk of BLV transmission and make it cheaper and easier to implement effective BLV countermeasures such as the isolation of high-risk cattle.

Our results clearly showed that, in the long term strategy for effective BLV eradication, measuring anti-BLV antibody titers can be used to estimate the risk of BLV transmission without evaluation of BLV PVL by real time PCR or determination of *BoLA-DRB3* alleles by PCR-SBT methods. In addition, it will allow for more efficient selective breeding of resistant dams and culling of susceptible dams to increase the number of BLV resistant dams on the farm. In the short term strategy for effective BLV eradication, measuring anti-BLV antibody titers can be used to reduce the risk of vertical transmission of BLV. We have previously reported that BLV PVL in milk and the infectivity of milk cells in resistant dams are lower than the ones in susceptible dams (48). This means that the risk of vertical infection *via* milk is lower in calves from resistant dams than that from susceptible dams. This provides the rationale for the new BLV vertical infection control measures that healthy dams should not be allowed to drink colostrum from susceptible dams. In addition, our results may also be applicable to field trials of vaccine efficacy. Recently, Suárez Archilla et al. reported that by inoculating Holstein dams with an attenuated live vaccine, most of them showed no wild-type BLV infection (54). As this study tested approximately 300 dairy cows over a 4-year period, it is better to use a large number of test cows over a long period of time in order to study the practical application of such a vaccine. Therefore, it is assumed that measuring the anti-BLV antibody levels in whey will allow for easier periodic monitoring of BLV infection status and BLV PVL. By contrast, non-lactating dams, calves, heifers, and bulls cannot be tested. These should be inspected as usual.

Previous reports have shown that anti-BLV antibodies in colostrum offer a certain degree of protection against BLV infection (30). The results of these studies suggest that colostrum from susceptible dams—which were thought to pose a higher risk of vertical infection—contains more anti-BLV antibodies. Vertical transmission through colostrum can be prevented by appropriate viral inactivation treatments such as heat treatment or freeze-thawing. Thus, feeding colostrum from properly treated BLV-infected and -susceptible dams is expected to provide calves with high protection against BLV infection during the first few months of life and may reduce the risk of vertical transmission *via* milk. By contrast, there are reports that show that colostrum containing anti-BLV antibodies poses a higher risk of vertical infection (28, 29). It is thus important to clarify the level of anti-BLV antibodies in colostrum that indicates protection against BLV infection.

## Data availability statement

The original contributions presented in the study are included in the article/supplementary material, further inquiries can be directed to the corresponding authors.

## Ethics statement

The animal study was reviewed and approved by RIKEN animal experiments committee (Approval Number H29–2–104). Written informed consent was obtained from the owners for the participation of their animals in this study.

## Author contributions

YA conceived of and designed the study. AN, LBo, LBa, MK, YM, JK, and YA collected the samples. AN, AB, SW, RM, and YA acquired, analyzed, and interpreted the data. AN, JK, and YA contributed reagents, materials, and analysis tools. AN and YA drafted and revised the manuscript. All authors agree to be accountable for the content of the work.

## Funding

This work was supported by grants from the Project of the NARO Bio-oriented Technology Research Advancement Institution (the special scheme project on regional developing strategy) (Grant No. 16817983) and by Grants-in-Aid for Scientific Research (A) from the Japan Society for the Promotion of Science (JSPS) (Grant No. JP16H02590).

## Conflict of interest

Authors AN and MK were employed by JA Zen-Noh. The remaining authors declare that the research was conducted in the absence of any commercial or financial relationships that could be construed as a potential conflict of interest.

## Publisher's note

All claims expressed in this article are solely those of the authors and do not necessarily represent those of their affiliated organizations, or those of the publisher, the editors and the reviewers. Any product that may be evaluated in this article, or claim that may be made by its manufacturer, is not guaranteed or endorsed by the publisher.
